# mySyntenyPortal: an application package to construct websites for synteny block analysis

**DOI:** 10.1186/s12859-018-2219-x

**Published:** 2018-06-05

**Authors:** Jongin Lee, Daehwan Lee, Mikang Sim, Daehong Kwon, Juyeon Kim, Younhee Ko, Jaebum Kim

**Affiliations:** 1 Department of Biomedical Science and Engineering, Konkuk University, Seoul, 05029 South Korea; 2 Division of Biomedical Engineering, Hankuk University of Foreign Studies, Yongin, 17035 South Korea

**Keywords:** Comparative genomics, Synteny block, Visualization

## Abstract

**Background:**

Advances in sequencing technologies have facilitated large-scale comparative genomics based on whole genome sequencing. Constructing and investigating conserved genomic regions among multiple species (called synteny blocks) are essential in the comparative genomics. However, they require significant amounts of computational resources and time in addition to bioinformatics skills. Many web interfaces have been developed to make such tasks easier. However, these web interfaces cannot be customized for users who want to use their own set of genome sequences or definition of synteny blocks.

**Results:**

To resolve this limitation, we present mySyntenyPortal, a stand-alone application package to construct websites for synteny block analyses by using users’ own genome data. mySyntenyPortal provides both command line and web-based interfaces to build and manage websites for large-scale comparative genomic analyses. The websites can be also easily published and accessed by other users. To demonstrate the usability of mySyntenyPortal, we present an example study for building websites to compare genomes of three mammalian species (human, mouse, and cow) and show how they can be easily utilized to identify potential genes affected by genome rearrangements.

**Conclusions:**

mySyntenyPortal will contribute for extended comparative genomic analyses based on large-scale whole genome sequences by providing unique functionality to support the easy creation of interactive websites for synteny block analyses from user’s own genome data.

## Background

Recent advances in next-generation sequencing technologies have significantly reduced the cost and time to sequence whole genomes of various species. Based on such technologies, many genome projects such as the Genome 10 K Project [1], the i5K project [2], and the bird 10 K project [3] have produced a huge amount of genome sequences, facilitating comparative genomics to identify genomic similarities and differences among different species and determine their functional consequences.

Synteny blocks are conserved genomic regions among species that play a pivotal role in comparative genomics. However, the construction of synteny blocks requires significant amounts of computational resources and time as well as bioinformatics skills necessary to prepare input data and specify various parameters for alignment of genome sequences. To address such difficulties, several web-based applications have been developed recently, including GRIMM-synteny [4], Cinteny [5], CoGe [6], and Genomicus [7]. However, functionalities and visualization methods of existing tools are not very satisfactory, especially for biologists who are unfamiliar with bioinformatics. To alleviate these problems, recently we have developed Synteny Portal [8], a versatile web-based application portal to construct, visualize, and browse synteny blocks in an intuitive and easy-to-use web interface. However, Synteny Portal is only available for a given set of genome sequences. In addition, it is not customizable for users who want to use their own sets of genome sequences or synteny blocks definitions.

To address these limitations, we developed mySyntenyPortal, a stand-alone application package to construct and publish websites for synteny block analysis. With mySyntenyPortal, users can easily build synteny blocks and construct websites for visualizing and browsing synteny blocks using their own genome sequences of multiple species or synteny block definitions generated using other tools.

## Implementation

**Fig. 1:**
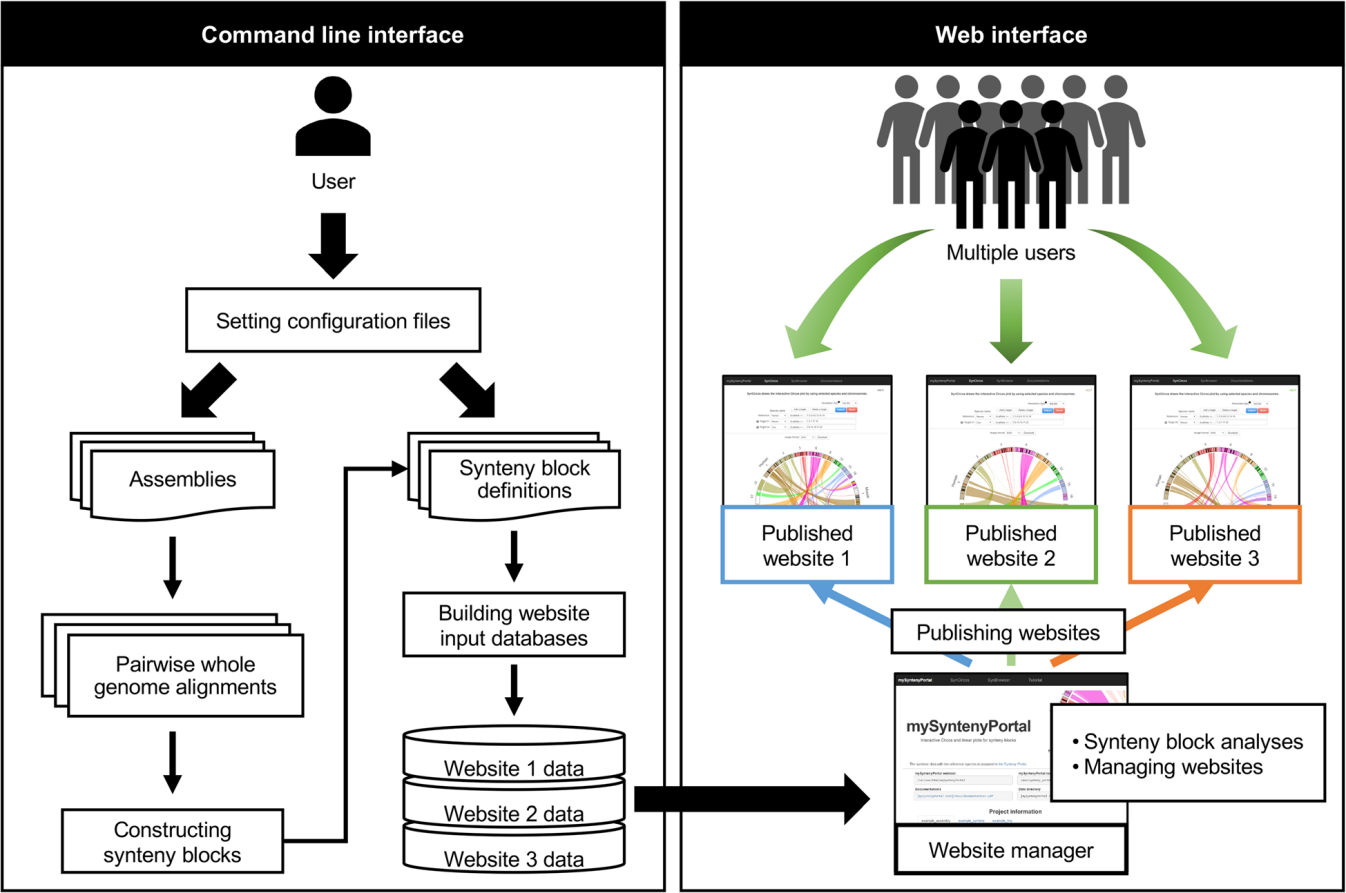
Workflow of using mySyntenyPortal. mySyntenyPortal consists of command line and web interfaces. User can build databases for synteny block analysis in the command line interface. In the web interface, users can perform analyses based on synteny blocks in built databases and publish websites for other users

mySyntenyPortal provides a command line interface to build a database for a website from user input data, and a web interface to manage and publish websites to make them available to other users (Fig. 1). Its command- line interface was implemented using Perl. Its web interface and published websites were implemented using HTML5, CSS, the Bootstrap framework (http://getbootstrap.com/), and JavaScript with libraries, such as jQuery (http://jquery.com), d3.js (http://d3js.org/), and GenomeD3Plot [9]. We note that mySyntenyPortal and Synteny Portal [8] are different applications because Synteny Portal is an *online website* for visualizing *prebuilt* synteny blocks, whereas mySyntenyPortal is an *offline standard-alone application* for *making* synteny blocks and source files for websites.

### Command-line interface

The command line interface provides a pipeline to build a database from user input data. For input data, user can specify genome assemblies of multiple species or synteny block definitions among multiple species with additional data, such as cytoband information and gene annotations. These cytoband information and gene annotations are used for visualizing cytobands in Circos plots and constructing gene annotation tracks, respectively. In the case when genome assemblies are provided as input, the pipeline can directly generate synteny blocks and use them to build the database. These synteny blocks are generated by two steps: (i) pairwise whole genome alignment, and (ii) synteny block construction. Pairwise alignment is performed by using LASTZ [10] and the Kent Utilities from the UCSC genome browser [11]. Synteny block construction is performed by using a modified module in the inferCars program [12]. In the case when synteny block definition is used as input, user needs to convert these definition files into a format supported by mySyntenyPortal. For convenience, the command line interface provides Perl scripts to convert synteny block definition files generated by three other tools, such as Cinteny [5], Satsuma [13], and SyntenyTracker [14], to unified synteny block definition files. Multiple independent databases can be created from different input data.

### Web interface

The web interface, called website manager, provides a unified management system to perform synteny-based analyses and manage/publish websites connected to these databases constructed using the command line interface. During synteny-based analyses, users can set a default state of a website being published to multiple users. The website manager also provides an interface to selectively publish or unpublish a website connected to a specific database. Once a specific database is published, webpages for this database are created with path information of these pages which can be used as URLs for public access.

### Published website

**Fig. 2:**
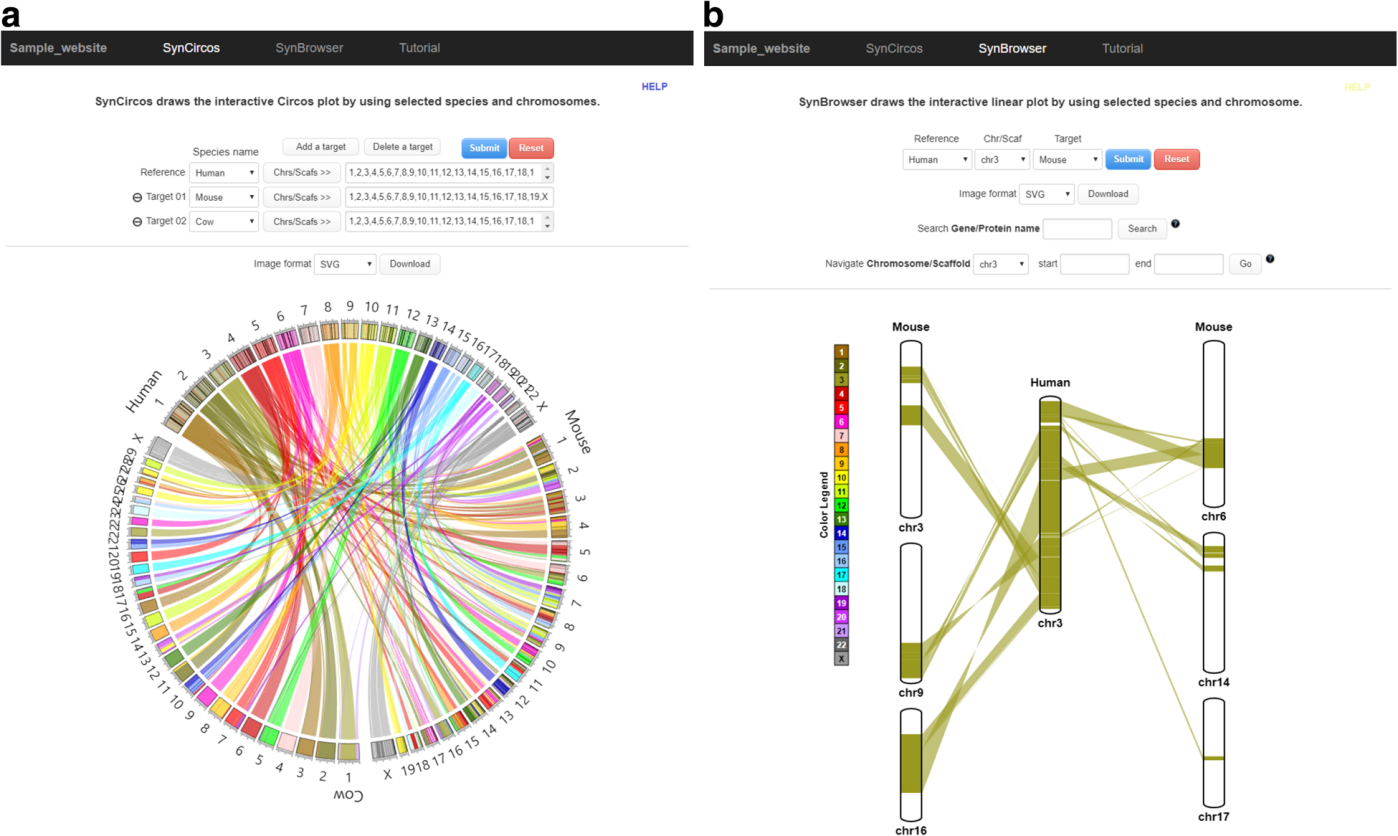
Example of a published website. **a** SynCircos page in the published website. **b** SynBrowser page in the published website

The website published by the website manager consists of two main web applications for synteny block analysis: SynCircos and SynBrowser. SynCircos produces an interactive Circos plot drawn by the Circos program [15]. SynCircos visualizes syntenic relationships between user-selected chromosomes (or scaffolds) of reference and multiple target species (Fig. 2A). Chromosome (or scaffold)-level relationships can be highlighted by placing a mouse pointer on it or clicking a specific chromosome (or scaffold). Cytobands can be optionally shown in the Circos plot.

SynBrowser makes linear plots to visualize syntenic relationships between a selected reference chromosome (or scaffold) and related chromosomes (or scaffolds) of a target species (Fig. 2B). An interactive gene annotation track can be optionally drawn by providing gene annotation information of the selected reference species. In the gene annotation track, users can easily browse the reference gene annotation track by mouse scrolling or dragging. Blue and green colored rectangles in the track represent plus and minus strand genes, respectively. Detailed information of annotated genes is shown when the mouse is pointed on the gene. In addition, user can directly access the UCSC genome browser [11] to obtain more details of genes by clicking the rectangular gene blocks. All plots generated by SynCircos and SynBrowser can be downloaded in various file formats, such as PNG, JPEG, PDF, and SVG. The published website can be simultaneously accessed by multiple users due to its session-based working environment.

## Results and discussion

### Building an example website for human, mouse, and cow

**Fig. 3:**
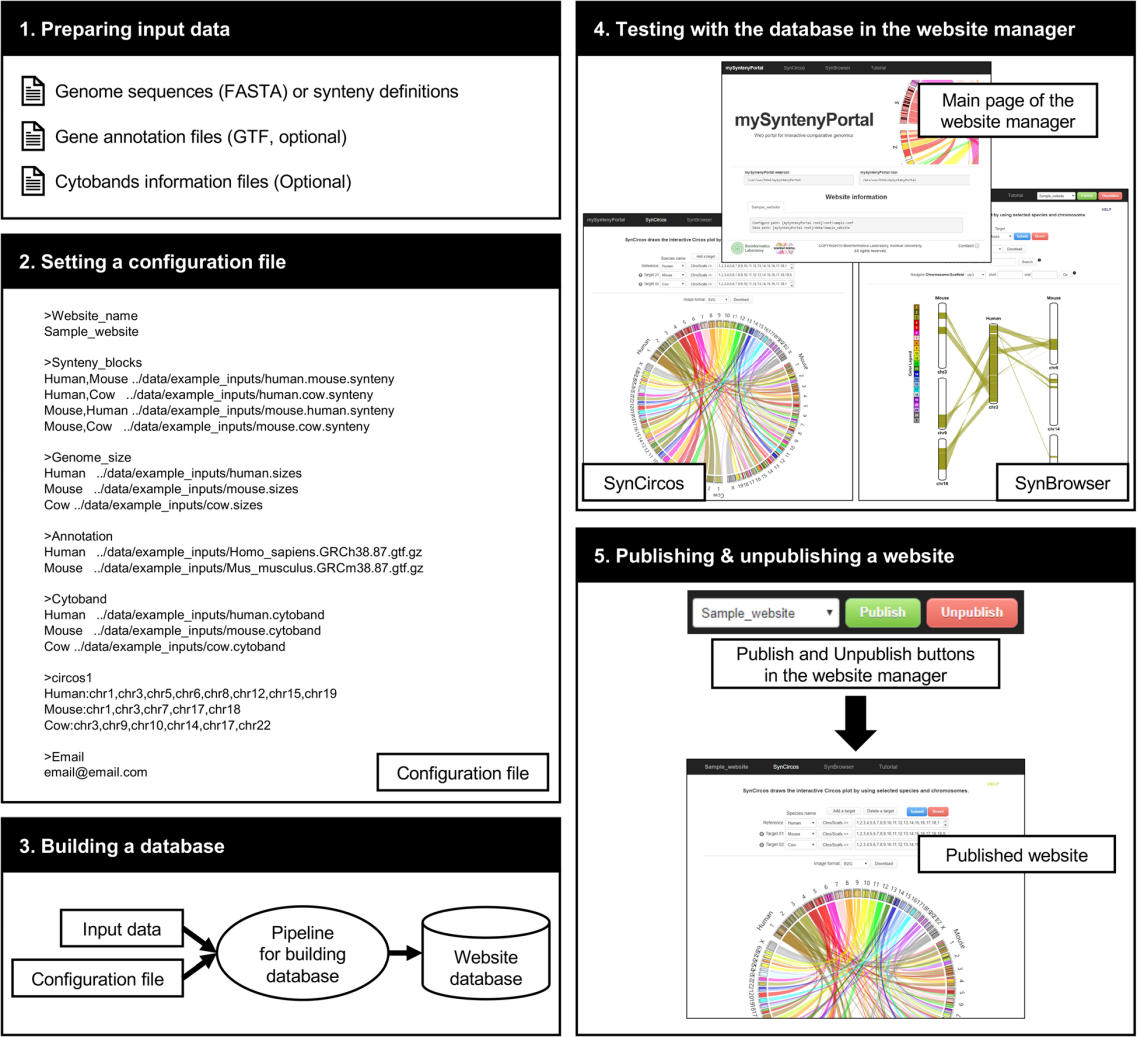
Example workflow for publishing a website for human, mouse, and cow synteny block analyses. User can publish a website for synteny block analyses through five steps: (1) preparing input data, (2) setting a configuration file, (3) building a database, (4) testing in the website manager, and (5) publishing a website

To demonstrate the usage of mySyntenyPortal, we analyzed genomes of three mammalian species: human (*Homo sapiens*, assembly GRCh38), mouse (*Mus musculus*, assembly GRCm38), and cow (*Bos taurus*, assembly Bos_taurus_UMD_3.1.1). Detailed workflow with data of these three species is shown in Fig. 3. Genome sequences and cytoband information of these species were downloaded from the UCSC genome browser. Gene annotations were obtained from the Ensembl genome browser [16] (Fig. 3, Step 1). A configuration file was then prepared with paths of genome sequence files, gene annotation files, cytoband information files, and parameter values to build a database (Fig. 3, Step 2). The pipeline for building a database was run with the configuration file (Fig. 3, Step 3). Once the database for these three species is created, additional information such as website name and paths of various files is provided in the main page of the website manager. At this point, synteny block analyses for the three species can be done through the SynCircos and SynBrowser modules before publishing the web interface to other users (Fig. 3, Step 4). The final website for other users can be also published by simply clicking the ‘Publish’ button in the website manager (Fig. 3, Step 5). The published website can be also unpublished by clicking the ‘Unpublish’ button.

### Synteny block analyses among human, mouse, and cow

**Fig. 4:**
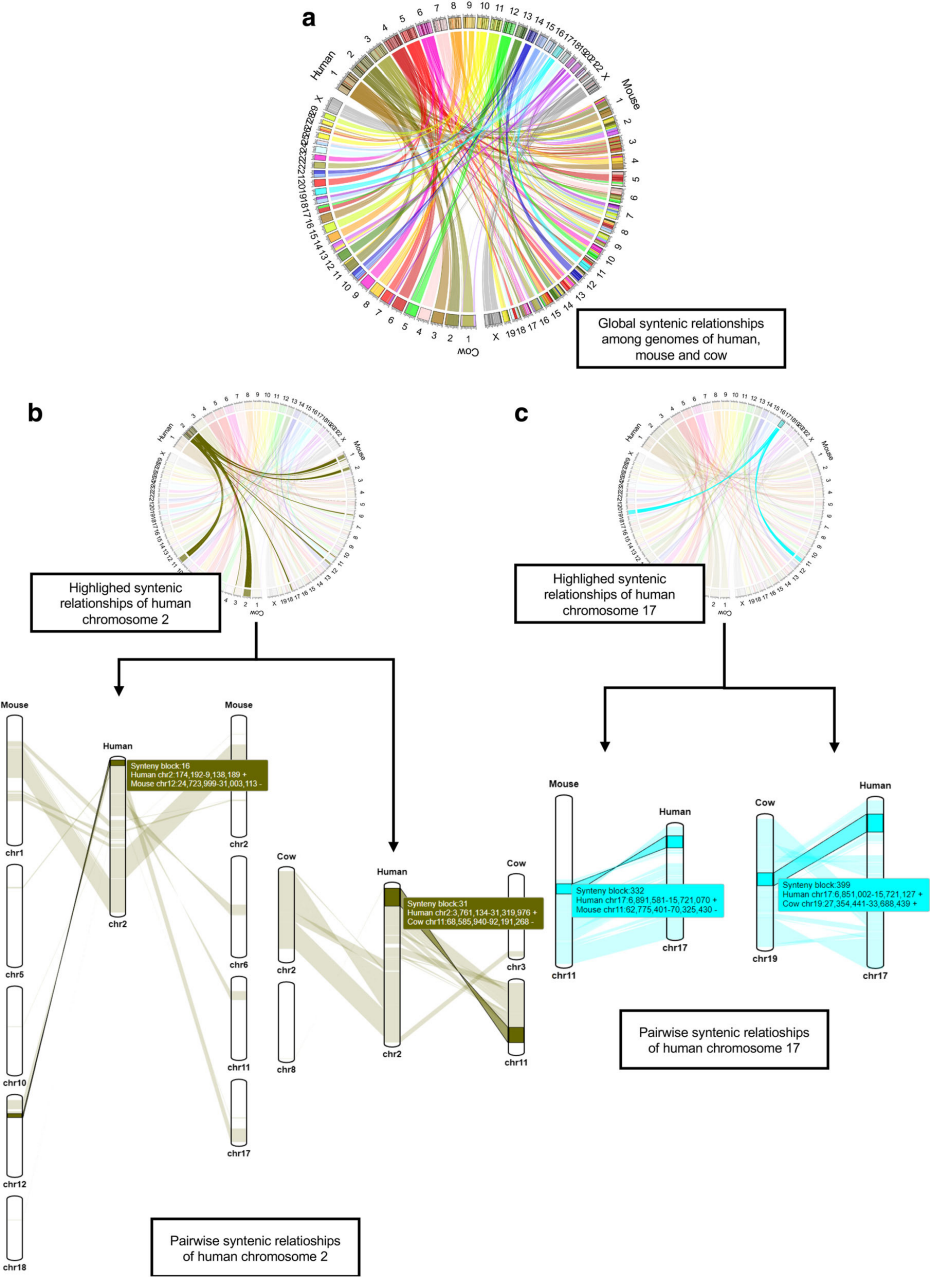
Examples of synteny block analyses using SynCircos and SynBrowser. **a** The Circos plot represents the syntenic relationships among the genomes of human, mouse, and cow. **b** The highlighted Circos plot and pairwise linear plots of synteny relationships of human chromosome 2 which has the maximum number of inter-chromosomal rearrangements. **c** The highlighted Circos plot and pairwise linear plots of synteny relationships of human chromosome 17 which has only intra-chromosomal rearrangements. In pairwise linear plots, coordinates of genomic regions belonging to each synteny block are provided in a floating box

With the SynCircos module of the website created by mySyntenyPortal, we visualized the global syntenic relationships among human, mouse, and cow with human as a reference species. Inter-chromosomal correspondence among these three species was easily identified from the Circos plot (Fig. 4A). Among all human chromosomes, chromosome 2 was found to have complex inter-chromosomal rearrangements compared to other species (Fig. 4B). A total of nine mouse chromosomes (1, 2, 5, 6, 10, 11, 12, 17, and 18), and four cow chromosomes (2, 3, 8, and 11) were connected to human chromosome 2. Detailed pairwise syntenic relationships could be detected in linear plots of the SynBrowser module (Fig. 4B). In these linear plots, exact chromosome locations involved in inter- and intra-chromosomal rearrangements could be obtained. On the contrary, human chromosome 17 showed only intra-chromosomal rearrangements with mouse and cow (Fig. 4C). The mode and extent of intra-chromosomal rearrangements were easily identified in the linear plots of the SynBrowser module.

**Fig. 5:**
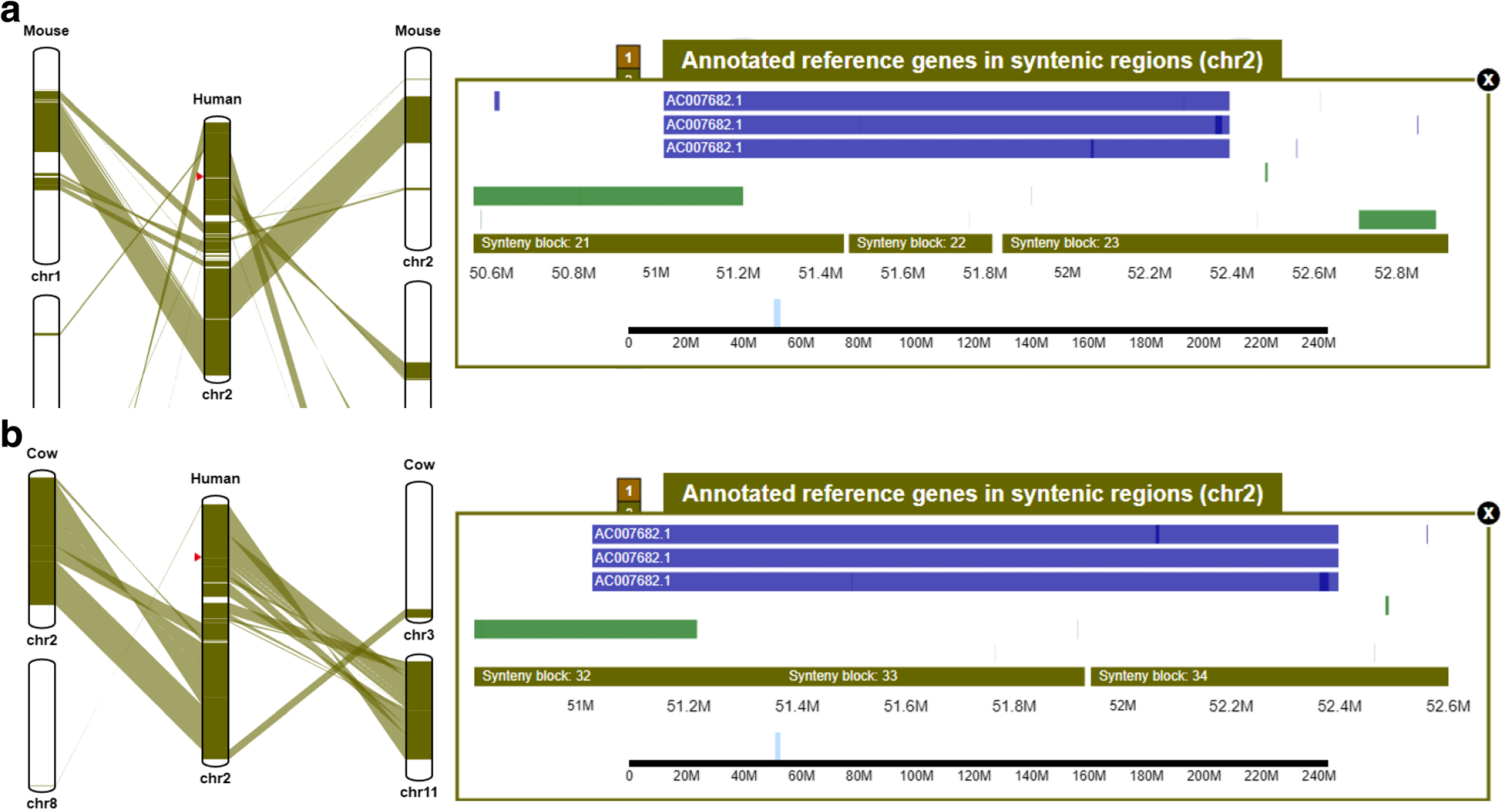
Examples of an annotated gene, AC007682.1, spanning multiple synteny blocks. **a.** Syntenic relationship with annotated genes between human and mouse. **b.** Syntenic relationship with annotated genes between human and cow. Red triangles in linear plots indicate positions of the annotated gene

The SynBrowser module also visualizes annotated genes of a reference species with their locations compared to synteny blocks (Fig. 5). This is very useful to identify conserved genes among multiple species (shown within a synteny block), genes specific to a reference species (shown between two synteny blocks), and genes broken by genome rearrangements (spanning multiple synteny blocks). For example, the human gene AC007682.1 spans three synteny blocks between human and mouse (Fig. 5A), and two synteny blocks between human and cow (Fig. 5B). These examples showed that human gene AC007682.1 might have appeared after speciation from cow and mouse.

## Conclusions

We developed mySyntenyPortal, which is an easy-to-use application to build and publish websites for synteny block analyses by using users’ own genome sequences or synteny block definitions. The published websites provide functionalities to visualize and browse synteny blocks effectively, which can support simultaneous accesses from multiple users. mySyntenyPortal will contribute to support extended comparative genomic analyses based on large-scale whole genome sequences by providing unique functionality to support easy creation of interactive websites for synteny block analyses using user’s own data.

## Availability and requirements

**Project name:** mySyntenyPortal.

**Project home page:** https://github.com/jkimlab/mySyntenyPortal

**Operating system:** Unix/Linux × 64.

**Programming language:** Perl, HTML, JavaScript and PHP.

**Other requirements:** Perl (5.10.1 or higher), Apache (2.2.15 or higher), ImageMagic (6.5.4 or higher).

**License:** GNU General Public License version 3.0 (GPLv3).

**Any restrictions to use by non-academics:** None.

## Abbreviations

CSS: Cascading Style Sheet
HTML: Hypertext Mark-up Language
